# *Azolla* along a phosphorus gradient: biphasic growth response linked to diazotroph traits and phosphorus-induced iron chlorosis

**DOI:** 10.1038/s41598-018-22760-5

**Published:** 2018-03-13

**Authors:** Ralph J. M. Temmink, Sarah F. Harpenslager, Alfons J. P. Smolders, Gijs van Dijk, Roy C. J. H. Peters, Leon P. M. Lamers, Monique M. L. van Kempen

**Affiliations:** 1 Aquatic Ecology and Environmental Biology, Institute for Water and Wetland Research, Radboud University, Heyendaalseweg 135, 6525 AJ Nijmegen, The Netherlands; 2 B-WARE Research Centre, Toernooiveld 1, 6525 ED Nijmegen, The Netherlands; 3 School of Biological and Chemical Sciences, Queen Mary University, E1 4NS London, United Kingdom

## Abstract

*Azolla* spp., a water fern often used for phytoremediation, is a strong phosphorus (P) accumulator due to its high growth rate and N_2_ fixing symbionts (diazotrophs). It is known that plant growth is stimulated by P, but the nature of the interactive response of both symbionts along a P gradient, and related changes in growth-limiting factors, are unclear. We determined growth, and N and P sequestration rates of *Azolla filiculoides* in N-free water at different P concentrations. The growth response appeared to be biphasic and highest at levels ≥10 P µmol l^−1^. Diazotrophic N sequestration increased upon P addition, and rates were three times higher at high P than at low P. At 10 µmol P l^−1^, N sequestration rates reached its maximum and *A. filiculoides* growth became saturated. Due to luxury consumption, P sequestration rates increased until 50 µmol P l^−1^. At higher P concentrations (≥50 µmol l^−1^), however, chlorosis occurred that seems to be caused by iron- (Fe-), and not by N-deficiency. We demonstrate that traits of the complete symbiosis in relation to P and Fe availability determine plant performance, stressing the role of nutrient stoichiometry. The results are discussed regarding *Azolla*’s potential use in a bio-based economy.

## Introduction

The genus *Azolla* comprises a broad range of floating aquatic fern species growing in tropical, subtropical and temperate freshwater ecosystems^1,2^. *Azolla* spp. are able to reach high growth rates by asexual reproduction and are very strong phosphorus (P) and nitrogen (N) accumulators^3^, which makes them very suitable for phytoremediation, bio-gas production, animal food and crop fertilization^1,4,5^. Due to their symbiosis with atmospheric nitrogen (N_2_) fixing microorganisms (diazotrophs), the primary production of the plants is hardly ever N-limited under natural conditions. The diazotrophs live inside *Azolla*’s leaf cavities (Supplementary Fig. S1) and include the cyanobacteria *Nostoc/Anabaena azollae*^6,7^ that forms unbranched, multi-cellular chains that contain both photosynthetic, vegetative cells and N_2_ fixing heterocysts^8^. Using nitrogenase enzymes, the diazotrophs reduce atmospheric N_2_ to ammonium (NH_4_^+^), which is then excreted into the *Azolla* leaf cavity and taken up by the fern^9^. In response to N-limitation, when there is no exogenous N available to *Azolla*, the heterocyst fraction increases in diazotroph chains^10,11^.

Given its high potential P uptake rates, luxury accumulation of P^12,13^ and high growth rates, *Azolla* may well be used for phytoremediation with respect to P^4,12,14–17^. The cultivation of *Azolla* spp. in P-enriched water could therefore offer a sustainable way of P recovery and recycling from eutrophic water^12,18–22^. Sustainable practices of P recycling are important, as global reserves of phosphate rock are becoming increasingly depleted^23,24^. Next to P recycling, *Azolla* cultivation may yield annually up to 35 t dry N-rich biomass, independent of N-fertilizer^25^. Although nutrient supply can easily be managed in hydro-cultures, nutrient management is more challenging when growing *Azolla* in flooded fields, due to biogeochemical interactions in the flooded soil and surface water^26^. The supply of macronutrients such as P and iron (Fe) and micronutrients by inflowing water, or by mobilization from the sediment to the overlying water should be sufficient to support *Azolla’s* growth^26,27^. Natural waters may well show non-optimal nutrient stoichiometry resulting in nutrient deficiency, impeded *Azolla* growth or even chlorosis^1,28^ due to P or Fe limitation^28,29^.

High P concentrations in surface waters are well known to stimulate the growth of *Azolla* spp.^30,31^ and to increase N_2_ fixation rates of their symbionts^31–33^. For efficient and broad application of *A. filiculoides* in a biobased economy, however, a profound understanding of the interactive responses of this species and its diazotroph microbiome to different P loadings is required, which is currently lacking. To test the type of growth response to P and find an explanation for this, *A. filiculoides* was grown in a greenhouse experiment in N-free surface water at different P concentrations. We hypothesized that both N and P sequestration rates are positively related to exogenous P concentrations, but level off because endogenous N provided by the diazotrophs becomes limited. We further hypothesized that at high P levels, Fe deficiency may occur which may induce chlorosis and limit *Azolla* growth, as this element is not only essential for *Azolla* but also for the production of the nitrogenase enzyme^1,28^.

## Results

### Growth response to P availability

**Figure 1:**
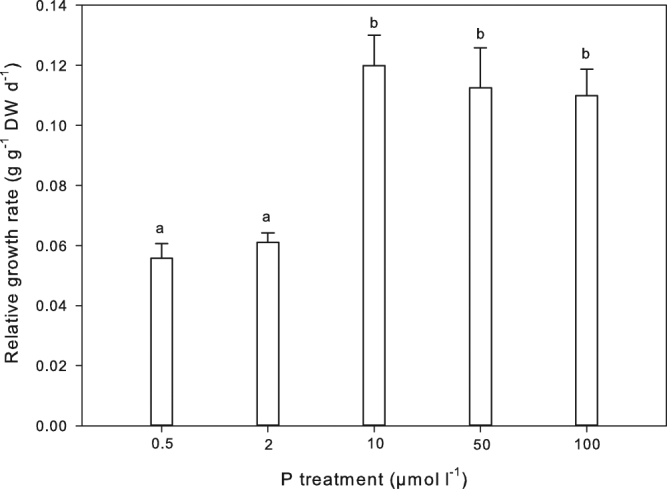
Relative growth rates (RGRs, g g^−1^ DW d^−1^) ± SE of *A. filiculoides* after 21 days of growth at 0.5, 2, 10, 50 and 100 µmol P l^−1^ (n = 5). Significant differences are indicated by different letters (Tukey HSD post hoc test). Additional results of the statistical analysis are presented in Supplementary Table S2.

RGRs of *A. filiculoides* growing in the 0.5 and 2 µmol P l^−1^ treatments were significantly lower than those of the higher P treatments (Fig. 1). *Azolla’s* growth response to increasing P showed a biphasic response (Michaelis-Menten fit: R^2^ = 0.62, p < 0.001). During 21 days, *A. filiculoides* growing in 0.5 and 2 µmol P l^−1^ treatments increased their biomasses 2.2 times, whereas in 10, 50 and 100 µmol P l^−1^ treatments, biomasses increased 3.4 times. During the experiment *Azolla’s* biomass was lower than the self-crowding threshold of 2 kg FW m^−2^ (data not shown).

Morphology of *A. filiculoides* also changed in response to different P treatments. Plants from the 0.5 and 2 µmol P l^−1^ treatments were smaller and more fragile than those from the 10, 50 and 100 µmol P l^−1^ treatments. Furthermore, plants from the lowest two concentrations turned reddish, while plants from the 10 µmol P l^−1^ treatment stayed green. Plants from the 50 and 100 µmol P l^−1^ treatments turned chlorotic (yellowish) after 21 days of growth.

### Plant nutrient composition

**Figure 2:**
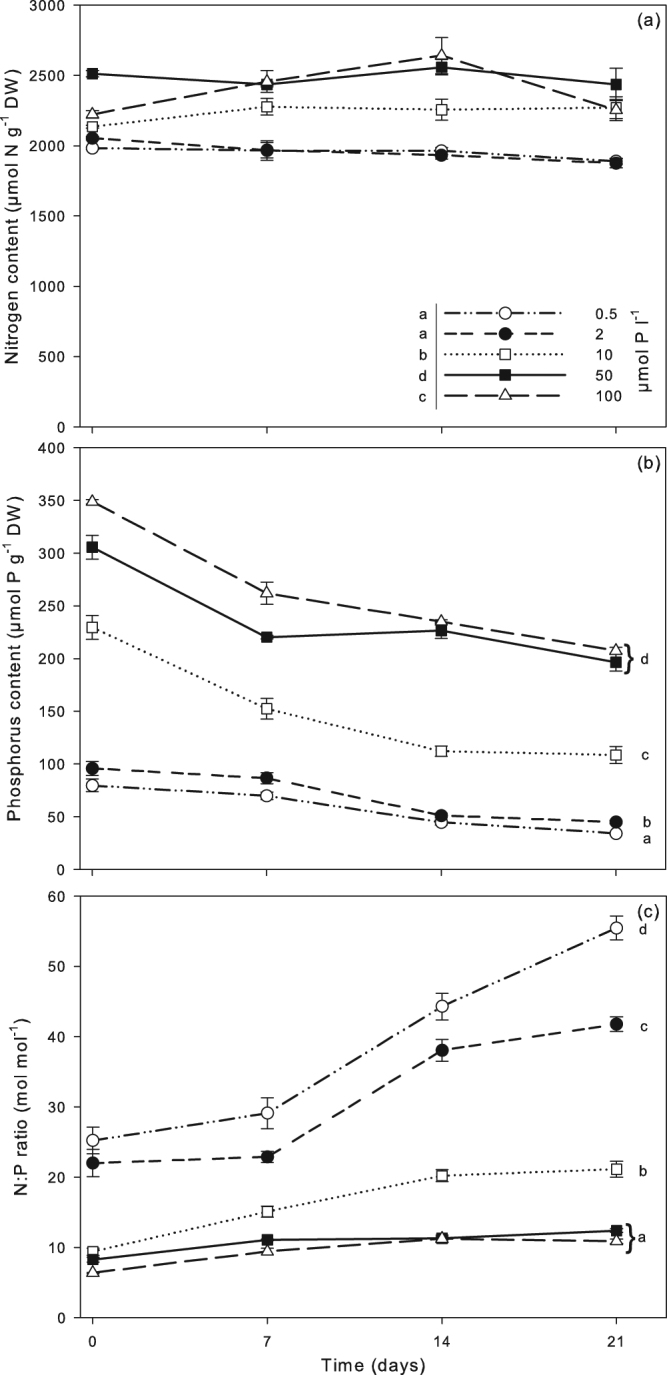
P content (µmol P g^−1^ DW; (**a**) N content (µmol N g^−1^ DW; (**b**) and N: P ratios (mol mol^−1^; (**c**) ±SE of *A. filiculoides* during 21 days of growth at 0.5, 2, 10, 50 and 100 µmol P l^−1^ (n = 5). Different letters indicate significant differences (Bonferroni post hoc test). Additional results of the statistical analyses are presented in Supplementary Table S2.

**Figure 3:**
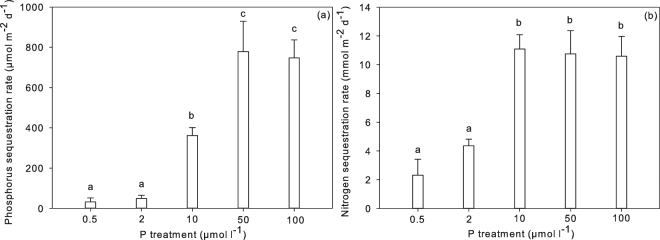
P (panel a) and N (panel b) sequestration rates (µmol m^−2^ d^−1^; n = 5) ± SE by *A. filiculoides* for the 0.5, 2, 10, 50 and 100 µmol P l^−1^ treatments. Different letters indicate significant differences (ANOVA Tukey post hoc test). Additional results of the statistical analyses are presented in Supplementary Table S2.

Plant P contents increased when P concentrations in the nutrient solution increased, but declined over time (Fig. 2a). P sequestration rates also increased, until the 50 µmol P l^−1^ treatment (Fig. 3a). Plant N content was stable over time, and highest at 50 µmol P l^−1^. Lowest N content was found for the 0.5 and 2 µmol P l^−1^ treatments (Fig. 2b). P concentrations ≥ 10 µmol l^−1^ in the nutrient solution significantly increased N sequestration rates of *Azolla* and its symbionts (Fig. 3b). N sequestration rates were approximately 4.5 and 2.5 times higher in the higher P treatments (≥10 µmol l^−1^) compared to the 0.5 and 2 µmol P l^−1^ treatments, respectively. Plant N: P ratios showed an extensive range, and decreased with increasing P availability (Fig. 2c). At day 21, the highest value (55 ± 1.7 mol mol^−1^) was reached in the 0.5 µmol P L^−1^ treatment, and the lowest in the 50 and 100 µmol P l^−1^ treatments (12.4 ± 0.3 and 10.9 ± 0.3 mol mol^−1^, respectively). Plant K, P, N, Na and S contents were significantly higher in the >10 µmol P l^−1^ treatments, whereas Zn and Ca contents were significantly lower and Fe and Si were similar (Supplementary Table S3). Further elemental composition of the plants is shown in Supplementary Table S3.

### Deficiency testing

**Figure 4:**
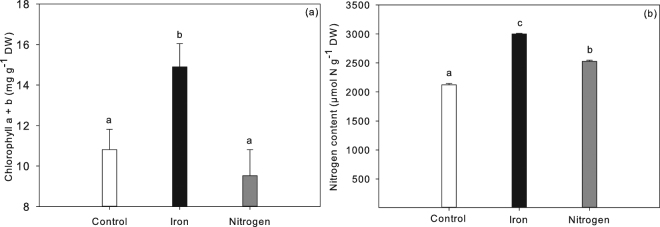
Chlorophyll a + b (mg g^−1^ DW, (**a**) and plant N content (µmol N g^−1^ DW, (**b**) of *A. filiculoides* treated with 100 µmol P l^−1^ (Control), after Fe (Iron) or nitrogen addition (limitation experiment, n = 4) ± SE. Significant differences are indicated by different letters (ANOVA Tukey HSD pairwise comparison). Additional statistical results are presented in Supplementary Table S2.

To test the cause of the observed chlorosis an additional, but similar, experiment was carried out, in which plants also turned chlorotic after 5 weeks of 100 µmol P l^−1^ treatment. They were subsequently treated with Fe or NH_4_^+^-NO_3_^−^. After two weeks, only the plants treated with Fe became green again and had a significantly higher chlorophyll a + b content (15.2 ± 2.3 mg g^−1^ DW) than the control (10.8 ± 2 mg g^−1^ DW) or the N treatment (9.5 ± 2.7 mg g^−1^ DW, Fig. 4a). In addition, highest N contents were found in plants treated with Fe (3000 ± 11 µmol N g^−1^ DW) and lowest in the control (2125 ± 20 µmol N g^−1^ DW; Fig. 4b). Plants treated with N showed an intermediate N content of 2529 ± 18 µmol N g^−1^ DW (Fig. 4b and Supplementary Table S4). Compared to both the control and the N treatment, the Fe content (55.8 ± 8 µmol Fe g^−1^ DW) in the Fe treatment was 1.6 and 1.5 times as high (Supplementary Table S4). Additional elemental composition of the plants is shown in Supplementary Table S4.

## Discussion

Both *Azolla filiculoides* growth and the diazotrophic activity of its microbiome (resulting in N sequestration) showed a biphasic response to P availability, when grown without external N supply. For high P levels, after maximum fixation rates by diazotrophic symbionts had been reached, the plants turned chlorotic. Additional external N supply did not increase plant health, but additional Fe supply did increase chlorophyll a + b and plant N contents. This not only proves that Fe was limiting at high P availability, but also means that traits of the complete symbiosis in relation to P and Fe availability determine *Azolla’s* performance, stressing the role of nutrient stoichiometry.

We found that *A. filiculoides* is able to double its biomass in one week in an N-free nutrient solution growing in a P-rich environment, entirely relying on the symbiosis with diazotrophs for its N supply. The growth rates measured are in the range reported in literature^32,34–37^. However, higher RGRs may be obtained when growing *Azolla* under optimal conditions using synthetic media in aquaculture^1,38^. Self-crowding may also result in lower RGRs of the fern, however, during the experiment the biomass was lower than the self-crowding threshold of 2 kg FW m^−2^ and was therefore not responsible for the biphasic growth response^39^. Here we show that the RGR of *A. filiculoides* shows a biphasic response to P availability, and becomes saturated at 10 µmol P l^−1^. The lack of differences between RGRs of plants grown with 10, 50 and 100 µmol P l^−1^, together with the substantially higher P content in plants grown at high P, either suggests that the maximum growth of this strain of *A. filiculoides* and its diazotrops were reached, or that a factor other than P became limiting above 10 µmol P l^−1^.

In aquatic plants, N: P ratios >17 (mol mol^−1^) generally indicate P deficiency, whereas a N: P ratios < 10 (mol mol^−1^) indicate N deficiency^40–42^. Based on their N: P ratios, *A. filiculoides* grown at P levels ≤ 2 µmol l^−1^ were severely P-limited^43,44^, which was also indicated by their red color^1^. Addition of P shifted the limitation through N and P co-limitation (in plants receiving 10 µmol P l^−1^) towards N-limitation (in plants receiving 50 and 100 µmol P l^−1^), based on N:P ratios and color.

**Figure 5:**
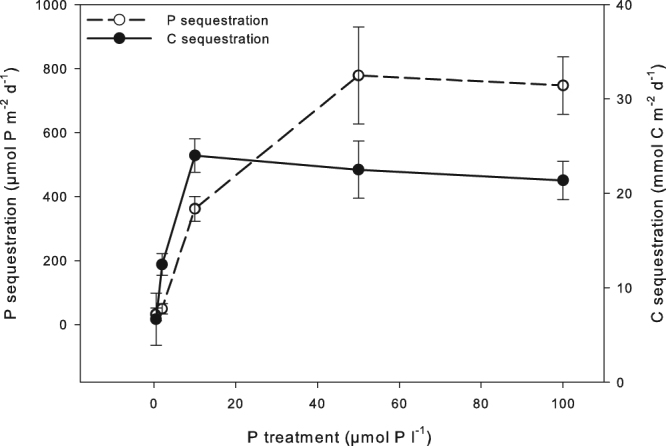
P (µmol P m^−2^ d^−1^; left y-axis) and carbon (C; mmol C m^−2^ d^−1^; right y-axis) sequestration rates in *A. filiculoides* under different P loadings (n = 15; ± SE).

*A. filiculoides* from the 50 and 100 µmol P l^−1^ treatment had a similar N: P ratio around 10 mol mol^−1^, which may indicate that the plant’s N-requirement and N-supply by the diazotrophs were in equilibrium to sustain *Azolla’s* growth. Apparently, the diazotrophs were not able to fix additional N that could be utilized to increase biomass growth. Interestingly, plants supplied with additional Fe had significantly higher N content compared to plants that were only supplied with N and plants that did not receive additional Fe or N, indicating Fe limitation for both plant growth and N_2_ fixation by the diazotrophs^1,25^. Luxury consumption of N did not seem to occur, probably because N_2_ fixation rates reached their maximum due to Fe limitation. The ferns, however, continued to accumulate P up to average rates of 778 µmol m^−2^ d^−1^, indicating luxury consumption (Fig. 5). N and P sequestration rates were indeed positively related to exogenous P concentrations. For N the maximum was reached at 10 µmol P l^−1^, whereas for P this was at 50 µmol P l^−1^. Our results clearly show that the diazotrophic community and *A. filiculoides* are able to respond dynamically to P availability in the aquatic environment, but become saturated or limited at a certain level.

In natural environments, eutrophication usually includes increased loading of both N and P. Presence of exogenous N influences both N_2_ fixation and growth of *A. filiculoides*. Availability of N, has been shown to inhibit nitrogenase activity in *Azolla*^11,15,45,46^, while not lowering plant growth^47^. In temperate regions, under natural conditions, surface water N concentrations rapidly decrease in spring as a result of high denitrification rates due to increased microbial activity, but surface water P availability remains high, especially if sediment P gets mobilized due to anoxia in the water layer^27,48^. Under these environmental conditions, *Azolla* can become highly competitive, using N produced by the diazotrophs combined with enhanced P availability.

At high P levels, plants may turn chlorotic, which has been attributed to absolute N-limitation in literature^1^. Here, we show that chlorosis and lower chlorophyll contents are caused by Fe deficiency at high P levels. Fe limitation can be expected to have resulted in lower growth and N sequestration rates in the high P treatments. The fact that *Azolla’s* condition (chlorophyll a + b) did improve after supplying exogenous Fe is in agreement with the hypothesis that Fe was deficient for *Azolla* grown at high P levels. In addition, it has been shown that *Azolla* is capable of higher growth rates when grown at >100 µmol P l^−1^ provided Fe is sufficiently available^2,32^. Fe availability in the environment or total Fe content of the plant are not the critical processes leading to chlorosis; multiple mechanisms can be responsible for Fe induced chlorosis including Fe precipitation in the apoplast, which is physiologically unavailable^49–51^. The presence of P can also affect plant Fe availability^44,52,53^. Interaction of P and Fe leading to Fe chlorosis can be caused by an internal immobilization of Fe probably due to formation of Fe-PO_4_^54–57^.

Our results indicate that nutrients in the water should be balanced when growing Azolla to optimize plant health and maximize yields. The use of *A. filiculoides* (and its diazotrophic community) as a tool to recover and recycle P in a bio-based economy^12,25^ is also dependent on Fe availability. When Azolla is grown on inundated agricultural lands (Azolla farming) for example, not only P re-mobilization from the sediment^27,48^, but also Fe mobilization should be considered.

## Materials and Methods

### Experimental set-up

Before the start of the experiment, *A. filiculoides* was acclimatized to experimental conditions in 15 l tanks containing an N-free nutrient solution to which 0.5, 2, 10, 50 or 100 µmol P l^−1^ was added (as NaH_2_PO_4_ * 2H_2_O). The general composition of the solution was similar to the quality of natural surface waters where *Azolla* is abundant (Supplementary Table S1). After sufficient *Azolla* biomass had been produced in the greenhouse for 30 days^37^, the plants were pretreated for 10 days in larger basins without harvesting to minimize P history effects^13^. Nutrient solutions were replaced 3 times a week. After this period^13^, 7.5 g (surface cover of 90%) of carefully dry-blotted *A. filiculoides* was transferred to a 1 l glass aquarium (10 cm × 10 cm × 12 cm; L × W × H; 750 g FW m^−2^), which was continuously fed by the same nutrient solution (pH = 7.3) as during the acclimatization period, at a rate of 1 l d^−1^ using peristaltic pumps (Masterflex L/S; Cole-Palmer, Chicago, IL, USA). Nutrient concentrations were checked biweekly. Each P treatment had 5 replicates. The water level was kept constant by an overflow outlet. Aquaria were randomly placed in a temperature controlled water bath at 18 °C situated in the greenhouse facilities, at a mean day temperature of 21.2 °C (SE ± 0.007) and night temperature of 18.6 °C (SE ± 0.007) during the experimental period of 21 days. Light was mostly natural, but an artificial light regime of 16 h: 8 h (light: dark) was maintained by four 400 W high-pressure sodium lamps (Hortilux-Schréder, Monster, The Netherlands) to illuminate the experiment whenever light intensity fell below 250 W m^−2^ (300 µmol m^−2^ s^−1^ PAR; Quantum sensor, Skye Instruments LTD, Wales, England). Sides of the aquaria were covered with black plastic foil to avoid light penetration from the sides.

### Growth measurement

Total plant biomass in each aquarium was determined at 0, 7, 14 and 21 days and subsamples were taken to determine fresh weight (FW) to dry weight (DW) ratios. Samples were rinsed, carefully blotted dry and weighed. After FW determination, 7.5 g (750 g FW m^−2^) of *A. filiculoides* was returned into its original aquarium to prevent crowding effects. The rest of the biomass was dried for 48 h at 60 °C, ground and homogenized using a ball mill (type Mixer Mill 301, Retsch GmbH, Haan, Germany) for further analyses. From the biomass increase, relative growth rates were calculated (RGR; g g^−1^ DW d^−1^).

### Plant nutrient analyses

200 mg dried plant material was digested using 4 ml HNO_3_ (65%) and 1 ml H_2_O_2_ (35%) in Teflon vessels using an Ethos D microwave (1200 MLS, Milestone, Sorisole, Italy), after which digestates were analyzed for P, Fe, aluminum (Al), manganese (Mn), sodium (Na), sulfur (S), silicon (Si), zinc (Zn) using an inductively coupled plasma emission spectrophotometer (ICP-OES; model IRIS Intrepid II XDL, Thermo Fisher Scientific, Franklin, USA). N contents of 3 mg homogenized dried plant material were determined by an elemental CNS analyzer (model NA 1500, Carlo Erba; Thermo Fisher Scientific, Franklin, USA). Plant N and P sequestration rates were calculated by combining plant biomass production and N and P contents for every harvest. As *A. filiculoides* was grown in an N-free medium, the increase in plant N could fully be attributed to N_2_ fixation by the diazotrophs.

### Iron versus nitrogen limitation experiment

To determine which element caused the chlorotic appearance of *A. filiculoides* found at 100 µmol P l^−1^, the original experiment was repeated for this treatment. *Azolla* may turn yellow/chlorotic due to the depletion of chlorophyll^1^. Also, Fe is often a limiting element for *Azolla* because it is an essential component of nitrogenase^1,28^. Chlorosis induced by iron deficiency, however, does not always implicate low total Fe contents in plant tissue^58–62^, because Fe may be immobilized in the apoplast and not be physiologically available^63,64^. Iron deficiency can be identified optimally when spraying plants with Fe, which causes regreening and an increase of chlorophyll a + b content^57,59,61,65^. To test N limitation, increasing both NO_3_^−^ and NH_4_^+^ in the medium is the optimal approach^45^. When *A. filiculoides* became chlorotic after 5 weeks, plants were daily treated with iron (sprayed with Fe-EDTA [*Pro Analysis quality*]; 10 µmol l^−1^) or nitrogen (NH_4_-NO_3_ [*Pro Analysis quality*]; 100 µmol l^−1^) for 2 weeks. Both methods were used to ensure optimal uptake of either Fe or N. To ensure that there would not be a time effect on the chlorotic appearance of *A. filiculoides*, the original treatment was continued (control, 100 µmol P l^−1^). Treatments were replicated 4 times. Nutrient content and chlorophyll a + b content^66^ were determined in all treatments at the end of the experiment, after 2 weeks of Fe and N application.

### Statistical analyses

Homogeneity of variance and normality of the residuals were tested, and Analyses of Variances (ANOVAs) were used to analyze potential differences in RGR, N and P sequestration rates and plant chlorophyll a + b in response to different P concentrations or to element addition (response chlorophyll a + b). Differences between treatments were determined using Tukey HSD post hoc tests. Data of P sequestration were square root transformed to authorize the use of parametric analyses. Linear mixed models (LMMs) were used to analyze differences in plant P and N content and N: P ratio over time at different P concentrations. Time was nested in aquaria and the covariance type was selected based on the smallest Hurvich and Tsai’s Criterion (AICC). P content or N: P ratio of the plants was set as a dependent variable while the P treatment was set as a fixed factor. Overall effects and P treatments were displayed in Estimated Marginal Means of Fitted Models and the main effects were compared using Bonferroni pairwise comparisons. Plant P content was log transformed and N: P ratios were square root transformed. All statistical analyses were carried out using SPSS 21.0 (SPSS Inc., Chicago, IL, U.S.A.).

### Data availability

The datasets generated during and/or analysed during the current study are available from the corresponding author on reasonable request.

## Electronic supplementary material

Supplementary information
