# Supplementary material for: Azolla along a phosphorus gradient: biphasic growth response linked to diazotroph traits and phosphorus-induced iron chlorosis
Source: Sci Rep. 2018 Mar 13;8:4451. doi: 10.1038/s41598-018-22760-5 (PMC5849758; doi:10.1038/s41598-018-22760-5)
Supplement: Supplementary file 1 — Supplementary information [file 41598_2018_22760_MOESM1_ESM.pdf]

Article title: *Azolla* along a phosphorus gradient: biphasic growth response linked to diazotroph traits and phosphorus-induced iron chlorosis

Authors: Ralph J.M. Temmink, Sarah F. Harpenslager, Alfons J.P. Smolders, Gijs van Dijk, Roy C.J.H. Peters, Leon P.M. Lamers, Monique M.L. van Kempen

Supplementary Information

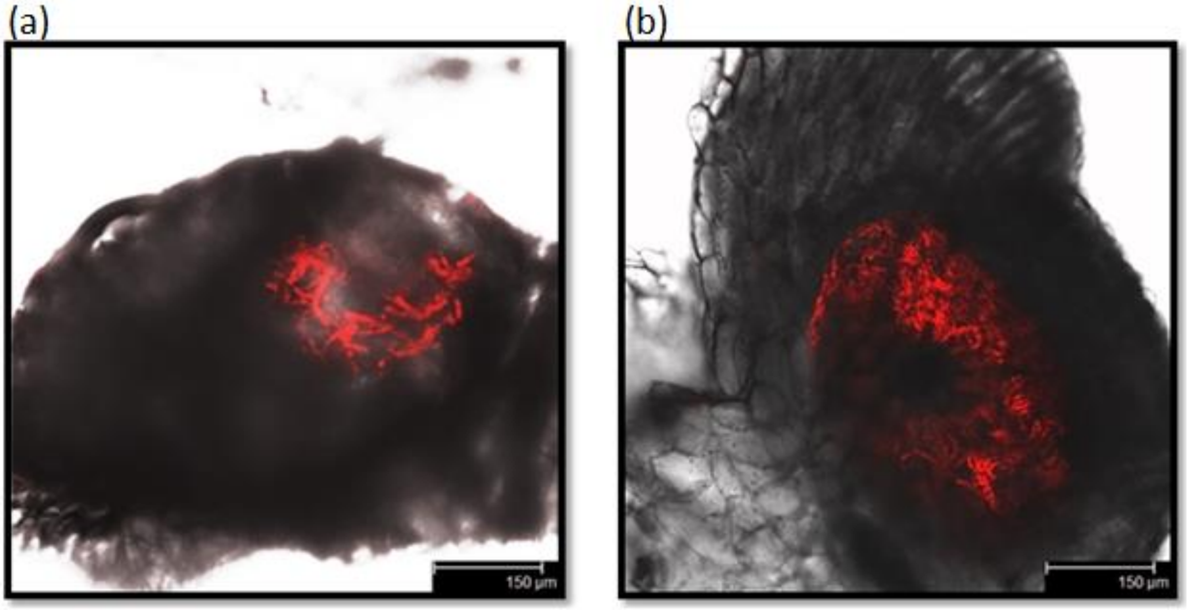

Supplementary Figure S1. CLSM image (overlay) of the lowest leaf (dorsal lobe) of *A. filiculoides* visualizing its diazotrophic cyanobacteria (red autofluorescence), grown at (a) 0.5 and (b) 100 µmol P l^{-1}. Grey cells represent photosynthetic cells. Diazotroph distribution in the *Azolla* leaf cavity was visualized using a Confocal Laser Scanning Microscope (CLSM; TCS SP2 AOBS, Leica-microsystems, Mannheim, Germany). The lowest leaf at the main axis was used, as this is known to host the highest number of diazotrophs (REF). A complete leaf was placed under a cover slip in Kaisers Glyceringelatine medium, and a 20x objective was used to create the images (excitation 488 nm at 51%, 496 nm at 41%, 514 nm at 40%, 561 nm at 55%; emission 577-671 nm; intensification 529 volt; pinhole 50-250 µm). Images were taken at different depths within the sample (z-stacks). The most detailed transmission image of the *Azolla* leaf and the most detailed autofluorescence image of the cyanobionts were combined using Adobe Photoshop CS6 (Adobe Systems Inc., San Jos, CA, U.S.A.). Picture: R. Temmink and L. Pierson.

Supplementary Table S1*. Chemical composition of the N-free nutrient solution, based on field conditions of natural stands (de Lyon & Roelofs, 1986). Different P treatments were created by adding 0.5, 2, 10, 50 or 100 µmol P l^{-1} (NaH_2PO_4 * $2H_2O$) to the medium.*

**Element**	**Concentration (µmol l^{-1})**
$NaHCO_3$	1000
$CaCl_2$*$2H_2O$	1000
K_2SO_4	500
$MgSO_4$*$7H_2O$	500
Fe-EDTA	10
$CuSO_4$*$5H_2O$	1
$MnCl_2$*$4H_20$	20
$ZnSO_4$*$7H_2O$	10
$NaMoO_4$*$2H_2O$	3
H_3BO_3	20
$CoCl_2$*$6H_2O$	4
NaCl	10

Supplementary Table S2. *Statistical results of the effects of 0.5, 2, 10, 50 and 100 µmol P l^{-1} (n=5) treatments for the relative growth rate (RGR; g g^{-1} DW d^{-1}), N : P ratio (mol mol^{-1}), N content (µmol N g^{-1} DW), P content (µmol P g^{-1} DW), P sequestration rate (µmol P m^{-2} d^{-1}), N sequestration rate (mmol N m^{-2} d^{-1}), chlorophyll a + b (mg g^{-1} DW; experiment 2) and N content (µmol N g^{-1} DW; experiment 2); ±SE. Significant differences are indicated by different letters.*

Relative growth rate (g g^{-1} DW d^{-1})						
	df	*F value*	*P*	Phosphorus	Tukey HSD pairwise comparisons	
ANOVA	4	12.072	<0.001	0.5	0.06 ± 0.005	a
				2	0.06 ± 0.003	a
				10	0.1 ± 0.01	b
				50	0.1 ± 0.01	b
				100	0.1 ± 0.009	b
N : P ratio (mol mol^{-1})						
	df	*F value*	*p*	Phosphorus	Bonferroni adjusted pairwise comparisons	
LMM (Covariance	4	733.53	<0.001	0.5	36.9 ± 0.6	a
				2	30.6 ± 0.5	b

type: unstructured)				10	16.1 ± 0.5	c
				50	10.5 ± 0.5	d
				100	9.3 ± 0.5	d
N content (µmol N g^{-1} DW)						
	df	*F value*	*P*	Phosphorus	Bonferroni adjusted pairwise comparisons	
LMM plant response (Covariance type: unstructured)	4	167.82	<0.001	0.5	1983 ± 19.4	a
				2	2040 ± 15.3	a
				10	2155 ± 15.3	b
				50	2510 ± 15.3	d
				100	2271 ± 15.3	c
P content (µmol N g^{-1} DW)						
	df	*F value*	*p*	Phosphorus	Bonferroni adjusted pairwise comparisons	
LMM plant response (Covariance type: unstructured)	4	69.273	<0.001	0.5	45.6 ± 3.3	a
				2	55.4 ± 3	b
				10	121 ± 3	c
				50	220.8 ± 3	d
				100	234.9 ± 3	d
P sequestration rate (µmol P m^{-2} d^{-1})						
	df	*F value*	*p*	Phosphorus	Tukey HSD pairwise comparisons	
ANOVA	4	38.549	<0.001	0.5	32.3 ± 20	a
				2	48.9 ± 16	b
				10	361.7 ± 39	c
				50	778.4 ± 151	d
				100	747.0 ± 90[c]	d
N sequestration rate (mmol N m^{-2} d^{-1})						
	df	*F value*	*p*	Phosphorus	Tukey HSD pairwise comparisons	
ANOVA	4	13.729	<0.001	0.5	2.3 ± 1	a
				2	4.4 ± 0.5	a
				10	11.1 ± 1	b

				50	10.8 ± 1.6	b
				100	10.6 ± 1.4	b
Chlorophyll a + b (mg g^{-1} DW) in experiment 2						
	df	*F value*	*P*	Treatment	Tukey HSD pairwise comparisons	
ANOVA	2	6.747	<0.02	C	10.8 ± 2	a
				Fe	15.2 ± 2.3	b
				N	9.5 ± 2.7	a
N content (µmol N g^{-1} DW) in experiment 2						
	df	*F value*	*P*	Treatment	Tukey HSD pairwise comparisons	
ANOVA	2	678.127	<0.001	C	2125 ± 20	a
				Fe	3000 ± 11	c
				N	2529 ± 18	b

Supplementary Table S3. *Results of the 0.5, 2, 10, 50 and 100 µmol P l^{-1} treatment (n=5) on the elemental content of A. filiculoides on day 21 (µmol g^{-1} DW) ±SE. Analyses of Variances (ANOVAs) were used to analyze potential differences in plant elemental contents. Differences between treatments were determined using Tukey HSD post hoc tests. Different letters indicate significant differences.*

		Phosphorus treatment (µmol P l^{-1})				
Elemental content (µmol g^{-1} DW)	*F value*	0.5	2	10	50	100
Al	19.01	1.8 ± 0.1^{b}	1.2 ± 0.1^{a}	0.9 ± 0.1^{a}	1 ± 0.1^{a}	1.04 ± 0.1^{a}
Ca	22.81	241 ± 3^{c}	199 ± 3^{b}	159 ± 7^{a}	159 ± 8^{a}	166 ± 12^{a}
Fe	0.982	26.3 ± 2^{a}	23.6 ± 1^{a}	25.1 ± 5^{a}	18.5 ± 2^{a}	21.9 ± 3^{a}
K	23.702	807 ± 15^{a}	965 ± 29^{b}	1252 ± 75^{c}	1179 ± 44^{c}	1132 ± 48^{bc}
Mg	8.786	142 ± 2^{c}	132 ± 3^{bc}	119 ± 4^{a}	129 ± 3^{ab}	130 ± 2^{ab}
Mn	3.016	145 ± 5^{b}	116 ± 5^{ab}	86 ± 10^{a}	115 ± 19^{ab}	106 ± 16^{ab}
Na	9.155	49.3 ± 1^{a}	55.5 ± 2^{a}	79.6 ± 7^{b}	103 ± 8^{bc}	87.6 ± 7^{b}
P	363.485	34.2 ± 1^{a}	45 ± 1^{b}	109 ± 6^{c}	196 ± 6^{d}	207 ± 3^{d}
S	7.298	139 ± 2^{a}	136 ± 2^{a}	162 ± 7^{b}	159 ± 5^{b}	154 ± 4^{ab}
Si	2.165	16.5 ± 2^{a}	18 ± 2^{a}	13 ± 2^{a}	11.1 ± 1^{a}	17.9 ± 3^{a}
Zn	41.706	37.4 ± 2^{b}	30.8 ± 2^{b}	18.6 ± 1^{a}	13.2 ± 1^{a}	15.6 ± 2^{a}
N	11.621	1890 ± 22^{a}	1875 ± 33^{a}	2270 ± 78^{b}	2436 ± 116^{b}	2254 ± 76^{b}

Supplementary Table S4. *Results of the Fe, N or no addition (n=4) on the elemental content of A. filiculoides grown at 100 µmol P l^{-1} after 2 weeks of nutrient addition (µmol g^{-1} DW) ±SE. Analyses of Variances (ANOVAs) were used to analyze potential differences in plant elemental contents. Differences between treatments were determined using Tukey HSD post hoc tests. Different letters indicate significant differences.*

Elemental content (µmol g^{-1} DW)	*F value*	Control	Iron	Nitrogen
Al	0.943	1.2 ± 0.1[a]	1 ± 0.1[a]	1 ± 0.04[a]
Ca	5.683	150 ± 7[a]	181 ± 8[b]	161 ± 5[ab]
Fe	4.598	35.8 ± 4[a]	55.8 ± 8[b]	37.9 ± 2[ab]
K	3.381	1309 ± 86[a]	1243 ± 34[a]	1095 ± 46[a]
Mg	2.039	112 ± 5[a]	111 ± 2[a]	103 ± 2[a]
Mn	2.349	107 ± 4[a]	133 ± 15[a]	149 ± 18[a]
Na	16.637	107 ± 5[b]	84 ± 1[a]	83 ± 3[a]
P	9.492	253 ± 12[a]	298 ± 4[b]	270 ± 6[b]
S	9.005	123 ± 8[a]	149 ± 3[b]	121 ± 4[a]
Si	1.334	5.6 ± 0.7[a]	5 ± 0.5[a]	4.5 ± 0.2[a]
Zn	1.569	27.2 ± 2[a]	25 ± 2[a]	30 ± 2[a]
N	678.127	2125 ± 20[a]	3000 ± 11[c]	2529 ± 18[b]
